# Knowledge, attitudes, and practices of healthcare professionals on oral care of pregnant women in Brunei Darussalam

**DOI:** 10.1038/s41405-023-00162-8

**Published:** 2023-07-31

**Authors:** Nur-Afifah Syafiqah Muhammad-Safwan, Khadizah H. Abdul-Mumin, Hanif Abdul Rahman, Rafidah Gharif, Haji-Mohin Haji-Momin, Ramlah Kisut, Ruth Zielinski, Jagjit Singh Dhaliwal

**Affiliations:** 1 Pengiran Anak Puteri Rashidah Sa’adatul Bolkiah Institute of Health Sciences, Universiti Brunei Darussalam, Gadong, Brunei Darussalam; 2 Department of Health Services, Ministry of Health, Bandar Seri Begawan, Brunei Darussalam; 3 Ministry of Health Dental Services, Brunei Darussalam, Bandar Seri Begawan, Brunei Darussalam; 4 Department of Nursing Services, Ministry of Health, Bandar Seri Begawan, Brunei Darussalam; 5 University of Michigan–Ann Arbor, Ann Arbor, MI USA

**Keywords:** Diseases, Health care

## Abstract

**Objective:**

To examine nationwide the knowledge, attitudes, and practices of healthcare professionals on oral/dental care of pregnant women in Brunei Darussalam.

**Materials and methods:**

A descriptive cross-sectional study conducted using an online survey of eligible healthcare professionals in all Government Maternal and Child Health Care Clinics, Dental Clinics and Obstetrics and Gynecological Clinics in four hospitals covering the whole of Brunei Darussalam. Participants were given seven days to complete the survey. Sub-group analysis using Chi-square test for independence and one-way Analysis of Variance (ANOVA) was used to determine the significant association between domains of oral care practices and demographic factors. *P* values less than 0.05 was considered statistically significant.

**Results:**

A total of 346 healthcare professionals participated in this online study. Most participants (94.3%) responded that they perceived oral health to be of high importance. However, less than two thirds of the participants (59.0%) included oral health questions during antenatal health assessments. Moreover, only 16.2% of participants frequently discussed the importance of oral care with their antenatal patients.

**Conclusions:**

In this survey, most healthcare professionals viewed oral health to be of high importance for prenatal/antenatal patients. However, not all put those views into practice with regard to oral care in pregnant women.

## Introduction

Oral health is an important aspect for maintaining the well-being of one’s general health [1]. The oral cavity is known to be a window to the overall health as it acts as an entry point into the body for organisms such as bacteria [2]. Hence oral health should be a priority for preventing illnesses, infections and oral diseases from occurring [3].

Maintaining good oral health is even more important for pregnant women as they are more vulnerable to developing oral diseases. This is due to the complex physiological changes alongside potential changes in eating patterns experienced throughout pregnancy which increases susceptibility to developing oral diseases such as dental caries, gingivitis, and periodontitis [4, 5]. Periodontitis, in particular, may not only bring harm to the health of the mother but may also have a negative effect on the pregnancy and the fetus [6–8]. There is an association between periodontal diseases and adverse pregnancy outcomes such as preeclampsia, low birth weight and preterm birth [8–10]. Therefore, it is very important for pregnant women to maintain good oral health and practice proper oral hygiene during pregnancy to prevent such complications from happening [11]. Pregnant women may not have adequate information regarding the importance of oral care and the complications associated with neglecting oral care during pregnancy [12]. Information and guidance from healthcare providers is an essential part of quality antenatal care [13].

For these reasons, healthcare professionals must have knowledge regarding assessment, education and treatment regarding oral care of pregnant women. Knowledge regarding oral care during pregnancy will increase attention toward the oral health of pregnant women and awareness of signs or symptoms which require further education, attention or dental referral [14]. An oral assessment of pregnant women by the healthcare professionals should be included during the initial antenatal appointments with ongoing assessments as needed [14]. If the pregnant women require dental attention, prompt treatment or referral is essential to restoring oral health and promoting optimal pregnancy outcomes. For these reasons healthcare providers play a very important role in ensuring pregnant women’s oral health is maintained [15]. Healthcare providers may not understand their role in maintaining oral health during pregnancy and may subsequently neglect oral health which puts their patients at risk of developing oral diseases [15].

This survey was conducted to provide insight into the healthcare professionals’ knowledge, attitudes, and practice regarding oral healthcare of pregnant women in Brunei Darussalam (henceforth: Brunei). Determining their level of understanding, attitude and current practice can be used to inform policy makers regarding prioritization of oral care during perinatal period.

## Materials and methods

### Study design

A nationwide quantitative and descriptive cross-sectional study was conducted through an online survey from August 2020 until May 2021 at the same time. All eligible participants, that is, healthcare professionals who provide care to pregnant women working in all the government maternal and child clinics, dental clinics, and obstetrics and gynecological clinics throughout Brunei, were recruited without sampling. With permission, a survey developed by Wilson et al. [16] was used with permission for data collection. The questionnaire was validated in the previous study, and therefore in the present study, only pretesting was done to ensure comprehensibility of the questions in this setting. The original survey tool was developed by content experts (dentists, obstetricians and a nurse-midwife). Content of the questionnaire was verified by experts in dentistry, medicine and midwifery in the research team. We also did face validity to ensure that the participants comprehension of the questions was consistent from one to another. There were no changes in the original instrument. We did this on five nurse-midwives. the tool is deemed to be culturally transferable to the Asian setting from the United States setting, in this case Brunei.

### Participant recruitment

Inclusion criteria included healthcare professionals (physicians, dentists, nurses, midwives and nurse-midwives) recruited from government maternal and child clinics in all four districts (Brunei-Muara, Tutong, Belait and Temburong); dental clinics; obstetrics and gynecological clinics in all four hospitals covering the nation of Brunei.

The following formula was used to calculate sample size for estimating a proportion of University students in an infinite (unknown) population.

$$n = \frac{{Z^2P\left( {1 - P} \right)}}{{d^2}}$$

Where, *n* = sample size, *Z* = *Z* statistic for a level of confidence, *P* = Expected prevalence or proportion, *d* = Precision

A sample size of at least 385 participants is required to achieve precision of 5% (*d* = 0.05) with expected proportion of 50% at 95% confidence level. Accounting for attrition and missing data, a 20% inflation was taken into consideration, therefore we distributed the survey to 500 healthcare professionals with the expectation that there would be 385 respondents (Table 1) [17].

**Table 1:** Sample calculation for different categories of healthcare professions.

Healthcare professionals	Study population	Expected minimum sample
Physicians	683 (18.1%)	90
Dentists	106 (2.8%)	14
Nurses	2713 (71.6%)	358
Midwives	289 (7.6%)	38
Total	3791	500

Figures are obtained from Ministry of Health Brunei Darussalam Health Information Booklet 2019 [26].

### Data collection method

Permission to conduct the study was obtained from the Director-General of Medical and Health services, Ministry of Health Brunei Darussalam. Following approvals, a meeting was held with the gatekeepers consisting of Director of Health Services, Director of Dental Services, Head of Maternal and Child Health Clinics, Head of Nursing Administration Community Health Nursing, Head of Obstetric and Gynecological Clinics, and Head of Nursing at Obstetric and Gynecological Clinics. They were briefed regarding the study and the procedure for recruitment of participants. The gatekeepers assigned officers to disseminate the survey link to all eligible healthcare professionals. Participants were given seven days to complete the survey. Two reminders were sent biweekly through the gatekeepers as to encourage higher responses and reduce non-response bias.

### Data analysis

All data processes were done using R studios software. A cleaning procedure was employed such as removal of ineligible cases, duplicate responses, and responses with more than 50% missing values (list wise deletion) to minimize errors in statistical analysis. A total of 481 responses were received and after data cleaning, 346 valid cases were used for analysis. Descriptive statistics were used to determine the level of knowledge, attitude and practice of healthcare professionals on the oral care of pregnant women throughout pregnancy. Sub-group analysis using Chi-square test for independence and one-way Analysis of Variance (ANOVA) was used to determine the significant association between domains of oral care practices and demographic factors. *P*-values less than 0.05 was considered statistically significant.

### Ethical considerations

This study followed the common ethical principles in a research. This study was approved by the PAPRSB Institutional Research Ethics Committee (UBD/PAPRSBIHSREC/2020/43). First, the decision to participate in the study was voluntary. Second, the confidentiality of the participants was protected as no personal data was collected in the questionnaire. Lastly, information regarding the purpose and aim of the study, along with the use of the data collected were included in the questionnaire in order to ensure informed consent was obtained.

## Results

### Socio-demographic characteristics

A total of 481 healthcare professionals were reached out and 346 completed responses in the survey were usable for analysis (response rate = 71.9%). The socio-demographic characteristics of the participants included age, gender, ethnicity, occupation, and years of work experience (Table 2). More than a third of the participants were of age ranging from 31 to 40 years old (39.5%). Most of the healthcare professionals that took part in the survey were females (91.8%) and were of Malay ethnicity (81.9%). Almost a third of the total number of healthcare professionals who participated were nurses (37.8%).

**Table 2:** Demographics characteristics of the healthcare professionals recruited.

Variables	*n*	%
Age (years) (*n* = 261)		
20–30	39	14.9
31–40	103	39.5
41–50	70	26.8
More than 50	49	18.8
Gender (*n* = 281)		
Male	23	8.2
Female	258	91.8
Ethnicity (*n* = 282)		
Malay	231	81.9
Chinese	10	3.5
Indian	3	1.1
Others	38	13.5
Occupation (*n* = 275)		
Physicians	31	11.3
Nurse	104	37.8
Midwife	49	17.8
Nurse-Midwives	66	24.0
Dentist	10	3.6
Dental Hygienist and Therapist	15	5.5
Work Experience (*n* = 262)		
<10 years	104	39.7
>10 years	158	60.3

**Table 3:** Factors associated with the healthcare professionals’ incorporation of dental/oral care in their prenatal/antenatal patients’ health assessments.

	*n* (%)
1. Does your prenatal/antenatal patient health assessment include questions regarding dental/oral health history? (YES)	204 (9.0)
2. If Yes to Question 1, how willing are you to add a question to your health assessment regarding patient dental/oral health history? [Willing/Very willing]	156 (9.1)
3. How often do you discuss with your patients the importance of dental/oral health as being a part of their prenatal/antenatal care?	
Always	72 (18.5)
Frequently	63 (16.2)
Sometimes	126 (2.3)
4. During the prenatal/antenatal visit/administration to the ward, do you look in your patient’s mouth for signs of dental/oral health problems? [Applicable for all respondents except for Dentists and Dental Hygienist and Therapist]	97 (26.2)
5. If Yes to Question 4, what helped you decide to do this? Please choose all that apply	
a) I know dental/oral health is an important component of prenatal/antenatal health care	108 (93.1)
b) I know dental/oral health is an important component for infant health	95 (91.3)
c) I could visibly see a dental/oral health problem	87 (83.7)
d) I have received training and feel competent to deliver a prenatal/antenatal dental/oral health	28 (27.7)
e) Dental/Oral health screenings are integrated into prenatal/antenatal care plans at the clinic where I practice	74 (73.3)
f) The patient mentioned a dental/oral health issue/problem	86 (78.2)
6. If No to Question 4, please tell us why you do not do this? Please choose all that apply	
a) I don’t believe it’s an important component of prenatal/antenatal health care	25 (13.7)
b) I do not have time to complete dental/oral health screenings during prenatal/antenatal care appointments	97 (51.3)
c) I have not received training to complete dental/oral health screenings	207 (85.5)
d) I do not feel competent completing dental/oral health screenings on my prenatal/antenatal patients	163 (74.4)
e) This procedure is not included in my scope of practice	212 (82.8)

(*n* = 346) Factors and factors associated with the healthcare professionals’ decision to look into prenatal/antenatal patients’ mouth to look for dental/oral problems [Applicable for all respondents except for Dentist and Dental Hygienist and Therapist]. (*n* = 321).

#### Table 3 shows results of participants’ inclusion of oral health history questions

More than half of the healthcare professionals (59.0%) responded that they did include oral health questions in the health assessment of their prenatal/antenatal patients. The top three healthcare professionals with the most prevalence of including oral health questions were dentists (87.5%), dental hygienist and therapists (72.7%), and nurses (58.1%). Approximately two-thirds of the participants had a working experience of over 10 years (64.0%). There was a significant association between age, occupation and year of work experience and the inclusion of oral health history questions in the health assessment of their prenatal/antenatal patients. In terms of age, there was significantly higher assessment of oral health history of prenatal/antenatal mothers for those over 40 years old (68.1% of those 41 to 50 years old vs. 42.0% of those 31 to 40 years old) (*p* < 0.001). In terms of occupation, the highest significance of the assessment of oral health history was by the dentists whereas lowest significance measured was by physicians (87.5% of dentists vs. 10.5% of physicians) (*p* = 0.001). In terms of year of working experience, a significantly higher assessment of oral health history was done by the healthcare professionals who had over 10 years of working experience (64.0% more than 10 years of work experience vs. 38.4% less than 10 years of work experience) (*p* < 0.001) (Table 4).

**Table 4:** Factors associated with oral health assessments for Dentist and Dental Hygienist and Therapist only (*n* = 25).

	*n* (%)
i. Please choose all that apply;	
a) I know oral health is an important component of prenatal/antenatal health care	19 (95.0)
b) I know oral health is an important component for infant health	19 (95.0)
c) I could visibly see an oral health problem	19 (95.0)
d) I have received training and feel competent to deliver a prenatal/antenatal oral health screening	15 (75.0)
e) Oral health screenings are integrated into prenatal/antenatal care plans at the clinic where I practice	15 (75.0)
f) The patient mentioned an oral health issue/problem	17 (89.5)
ii. Please choose all that apply;	
a) I don’t believe it’s an important component of prenatal/antenatal health care	1 (5.0)
b) I do not have time to complete oral health screenings during prenatal/antenatal care appointments	3 (15.0)
c) I have not received training to complete oral health screenings	2 (10.0)
d) I do not feel competent completing oral health screenings on my prenatal/antenatal patients	2 (10.0)
e) This procedure is not included in my scope of practice	8 (40.0)
iii. How often do you get referrals of prenatal/antenatal patients?	
Frequently	1 (5.0)
Sometimes	11 (55.0)

#### Participants’ willingness to add oral related questions

Most of the participants (89.1%) responded that they were willing to add questions regarding oral health to their health assessments. The top three healthcare professionals who responded with their willingness to add oral health questions were physicians (100.0%), midwives (100.0%), and nurses (94.1%). Though, no significant association had been identified between these variables with the willingness to add oral health questions.

#### Table 3 shows participants’ frequency of discussing the importance of oral health in prenatal/antenatal care

A third of the healthcare professionals (32.3%) responded that they discussed oral health ‘sometimes’, 16.2% responded ‘frequently’, 18.5% responded ‘always’ and the remaining 33% responded ‘never’. The results from the responses of healthcare professionals on sometimes discussing with the prenatal/antenatal patients was significantly associated with age, occupation and year of working experience. In terms of age, a significantly higher association was for those over 40 years old (45.5% of those 41 to 50 years old vs. 25.0% of those more than 50 years old) (*p* < 0.001). In terms of occupation, there was significantly higher frequency of discussion done by the nurse-midwives (47.8% of nurse-midwives vs. 0.0% dentists) (*p* < 0.001). In terms of year of working experience, those who had over 10 years of working experience were more likely to discuss oral health compared to those who had less than 10 years of work experience (40.6% > 10 years of work experience vs. 25.3% < 10 years of work experience) (*p* < 0.001).

#### Participants’ practice of performing an oral exam

Over a quarter of the healthcare professionals (26.2%) responded that they looked into their patients’ mouth for any signs of oral problems. One quarter of nurse-midwives reported performing an oral exam (25.0%) whereas 13% of physicians reported looking into their patients’ mouths although this different was not statistically significant. In terms of age, participants over age 50 were more likely (36.8%) to perform and oral exam than those who were 20 to 30 years old (16.1%, *p* = 0.016). In terms of year of work experience, participants who had over 10 years of working experience were more likely to perform an oral exam (23.4%) versus those with less than 10 years of work experience (15.1%, *p* = 0.003) (Table 3).

#### Participants’ reason for performing an oral/dental exam

Almost three-quarters of the healthcare professionals (73.3%) responded that the oral health integrated into screening for antenatal patients at their clinic was the most common reason for performing an oral exam. Of the different professions, nurses were most likely to indicate screening as the reason for an oral exam and regarding experience, those with more than 10 years of work experience (73%) were more likely to indicate this as the reason although these differences did not reach statistical significance. There was significant association between participant age and screenings as the reason for performing an oral exam. Healthcare professionals aged more than 40 years old had more oral health screenings at their clinic (83.3% of those 41 to 50 years old vs. 20.0% of those 20 to 30 years old) (*p* = 0.041) (Table 3).

Most participants responded with not being trained as being the reason for not performing oral health screenings or exams (85.5%). Not believing that oral health was an important component of prenatal care was the least chosen reason for non-performance oral health screenings (13.7%). Time was an additional factor with 51.3% of respondents indicating not having the time to do oral health screenings during antenatal appointments. There was a significantly higher number of healthcare professionals who were more than 50 years of age that responded with not having the time (81.8% vs. 39.1% of those 41 to 50 years old) (*p* = 0.034) (Table 3).

#### Participants’ frequency on receiving referrals

More than half of the dental professionals (55.0%) responded that they sometimes received referrals of antenatal patients. A majority of the dental professionals (80.0%) who responded ‘sometimes’ had more than 10 years of working experience (Table 5). There was no significant association between the frequency of receiving referrals with years of work experience.

**Table 5:** Factors associated with referrals of antenatal patients.

	n (%)
I. During the antenatal visit/admission to the ward, do you currently refer your patients to a dentist if an oral health problem is identified?	
Yes	210 (54.3)
Sometimes	51 (13.2)
II. If Yes to Question 7, what helped you decide to do this? Please choose all that apply	
a) There is a list of Oral Clinics that accept referrals of pregnant women requiring oral health care	164 (85.0)
b) Referring patients to an oral clinic is a professional obligation	172 (88.2)
c) I work in a clinic/ward that is equipped to provide oral health care	103 (56.0)
d) Professional oral health care is integrated within my patients prenatal/antenatal care plan	141 (78.8)
III. If No to Question 7, please tell us why you do not currently refer your prenatal/antenatal patients to a dentist if a oral health problem is identified. Please choose all that apply	
a) I do not have referral arrangements with dentist(s) in my clinic/ward	85 (73.9)
b) The referral process is too time consuming	40 (42.6)
c) There is not enough time during a prenatal/antenatal care visit to discuss oral referral options	57 (58.8)
d) This is not my professional obligation	92 (76.7)
IV. How often do you refer your prenatal/antenatal patients to a oral provider if they are not receiving oral care? [Applicable for all respondents except for Dentist and Dental Hygienist and Therapist]	
Always	40 (11.0)
Frequently	47 (13.0)
Sometimes	132 (36.5)

#### Table 5 describes participants’ practice of referring their patients to the dentist

More than half of the participants (54.3%) responded that they did refer their antenatal patients to the dentist when they identified oral problems. Of the healthcare professionals who referred patients 69.6% were physician, 51.1% were nurses, and 50% were midwives (50.0%), with no significant association between referring and occupation. 13.2% of healthcare professionals responded that they sometimes referred their prenatal/antenatal patients if they identified oral problems. There was significant association between this decision with age, occupation, and year of work experience. In terms of age, there was a significantly higher association of this option with those aged between 20 and 30 years old (26.5% of those 20–30 years old vs. 8.8% of those 41–50 years old) (*p* = 0.007). In terms of occupation, the top three healthcare professionals who were of high significance were dentists (37.5%), nurse-midwives (21.3%) and physicians (17.4%) (*p* = 0.029). In terms of year of work experience, there was a significantly higher association of referring the prenatal/antenatal patients with those who had less than 10 years of work experience (18.7% of those less than 10 years of work experience vs. 11.0% of those more than 10 years of work experience) (*p* = 0.007).

#### Participants’ frequency of referring their patients for dental care

More than a third of the healthcare professionals (36.5%) responded that they sometimes referred their antenatal patients who were not receiving oral care to the dentist. While more nurse-midwives (43.1%) reported referring patients, the difference did not reach statistical significance. Participants between 41 and 50 years of age were more likely (46.3%) to refer patients than those who were older (22.5%, *p* = 0.040) (Table 5).

#### Participants’ reason for referring their patients

Most healthcare professionals who referred antenatal patients for dental care (88.2%) responded that it was a professional obligation to refer antenatal patients for dental care. Access to a list of oral clinics that accepted pregnant women was also important for referrals (85.0%). Respondents who chose this option as their reason were dental hygienists/therapists (100.0%), nurses (91.3%), and midwives (84.0%). In terms of years of work experience, those with over 10 years of work experience (91.7%) were more likely to indicate a list of referral options as a reason than those with less experience (67.7%, *p* = 0.005).

#### Participants’ reason for not referring their patients

Of respondents who did not refer patients, more than three-quarters (76.7%) responded that referring pregnant patients for dental care was not part of their professional obligation. Of participants that did not refer patients, 73.9% responded that they did not have referral arrangements (Table 5).

#### Participants’ professional referral arrangements

Only 15.7% of healthcare professionals responded that they had established professional referral arrangements. Providers who were more than 50 years old (35.6%) were more likely to have a professional arrangement than those who were in the youngest age bracket of 20 to 30 years old (5.7%. *p* = 0.003).

#### Participants’ collaboration/engagement in professional discussions

More than a third of the participants (37.4%) responded that they had collaborations and did engage in professional discussions with other healthcare professionals for the care of a mutual patient. The top three healthcare professionals who engaged in collaborations and discussions were dentists (66.7%), nurses (55.6%), and nurse-midwives (40.0%). Less than two-thirds (60%) of the healthcare professionals who collaborated responded that they did so at least once a year (Table 6).

**Table 6:** Factors associated with the collaboration between healthcare professionals on the oral care of prenatal/antenatal patients (*n* = 346).

	*n* (%)
I. Do you have professional referral arrangements established with local dentist(s) to whom you can refer your antenatal patients? [Answer options combined – 1 and 1/more]	61 (15.7)
II. If Yes to Question 11, do you collaborate or engage in professional discussions with referring dentist(s) regarding care for a mutual patient?	43 (37.4)
III. If Yes to Question 12, how often do you collaborate or engage in professional discussions with referring dentist(s) regarding care for a mutual patient	
1x per year	39 (60.0)
2–4x per year	15 (23.1)
More than 4x per year	11 (16.9)
IV. Are you interested in receiving additional information to enhance your knowledge regarding prenatal/antenatal oral healthcare?	274 (71.5)
V. Please tell us how and/or what type of format you would like to receive information regarding prenatal/antenatal oral healthcare	
a) I would attend a Continuous Professional Development (CPD) course(s), such as a one-day workshop or seminar that is focused on current best practices regarding prenatal/antenatal oral healthcare	246 (78.8)
b) I would prefer to read information/research articles on current best practices regarding prenatal/antenatal oral healthcare	63 (20.2)
VI. Are you interested in attending interprofessional collaboration activities such as professional networking, Continuous Professional Development (CPD) course(s), and meetings etc. with oral healthcare providers in your clinic/ward?	293 (5.1)
VII. “I consider oral health care to be an important part of a prenatal/antenatal care plan.”	366 (94.3)

#### Participants’ attitude towards enhancing their knowledge

A majority of the respondents (71.5%) indicated that they were interested in continuing education regarding antenatal oral care. Healthcare professionals that had the highest prevalence of interest were dentists (100.0%), physicians (86.4%), and nurse-midwives (78.0%) (Table 6).

#### Participants’ preferred format to enhance their knowledge

More than three-quarters of the healthcare professionals (78.8%) responded that they would prefer to attend a Continuous Professional Development (CPD) course whereas only 20.2% of the respondents preferred to gain the information through reading or research articles (Table 6).

#### Participants’ interest on interprofessional collaboration activities

The majority of healthcare professionals (75.1%) responded that they were interested in attending interprofessional collaboration activities with oral healthcare providers. Healthcare professionals that had the highest prevalence of interest were dental hygienist/therapists (83.3%), physicians (81.8%) and nurse-midwives (81.7%). Most of the healthcare professionals had work experience of less than 10 years (81.9%) (Table 6).

#### Participants’ attitude towards importance of oral health in prenatal/antenatal care

Most healthcare professionals (94.3%) considered oral healthcare to be an important part of the antenatal care plan. Healthcare professionals that considered this important were dentists (100.0%), dental hygienist and therapists (100.0%), and midwives (97.8%) although the difference between professions was not significant (Table 6).

## Discussion

The objective of this descriptive survey was to explore healthcare professionals’ knowledge, attitudes and practice regarding oral/dental care of pregnant women. Through assessing knowledge, attitudes and practice, we can gain insight the current situation regarding oral care of pregnant women and where improvements can be made. While a majority responded that they included oral health as part of their assessment, only a quarter of respondents looked into their patients’ mouth to assess for any signs of oral problems. Previous studies reported similar results in that fewer than one-quarter of prenatal health professionals looked into patients’ mouth during an ante natal examination despite the importance of oral health to the care of pregnant women [18]. Oral examination is particularly important as it helps in the early identification and treatment of oral disease during pregnancy that could impact perinatal outcomes. This importance was highlighted in another study where they emphasized the importance of not delaying necessary oral care as it may bring harm to both the mother and fetus [19].

While just over half of healthcare respondents in this study currently included oral health questions in the assessment of antenatal patients most indicated a willingness to add oral health questions. This indicates that most of the healthcare professionals who responded to this survey have a positive attitude towards including oral care for antenatal patients even if they were not currently addressing oral health. Similar findings were reported in another study with a majority of respondents expressing interest in gaining knowledge in order to provide dental assessments for antenatal patients [20].

While healthcare providers understood the importance of oral/dental care during pregnancy, few indicated that they always discussed it with patients. Healthcare professionals play a crucial role in the education of antenatal patients as are the primary source of evidence-based information during pregnancy. A prior study found that the majority of respondents chose health professionals as their main source of knowledge regarding oral care [21]. Therefore, educating and discussions with patients regarding oral care is a very important component of antenatal care.

Performing an oral/dental examination by the healthcare professional is an essential component in the assessment of the oral condition of antenatal patients [19]. In this survey, respondents indicated that not receiving training in oral health screenings was a primary reason for not looking into patients’ mouth. This result ties well with another study Other studies of barriers to perinatal oral health practices reported similar findings in that antenatal care providers identified insufficient training as being a barrier to performing oral examination for the prenatal/antenatal patients [20].

Pregnant women should be referred to the dental clinic for appropriate evaluation to help in early management and intervention of oral health issues the pregnant women might be experiencing in order to reduce risk to mother and fetus [22]. In this study, referrals during pregnancy were infrequently provided indicating a lack of healthcare professionals who provide referrals for their prenatal/antenatal patients. In another study assessing the health professionals’ evaluation of oral care in pregnant women, guidelines health professionals can take in order to implement a more multidisciplinary approach towards the oral care of pregnant women including referrals for treatment [18]. This further emphasizes the importance of referring antenatal patients for appropriate oral care.

Healthcare professionals’ attitude towards oral/dental care of pregnant women can be highlighted by the low rate of referral with just slightly over half of the respondents in this study indicating that they referred patients once they identified any oral problems. Even less referred their patients who did not have a problem but were not receiving oral care. Those who did not give referrals did not think it was a part of their professional obligation. In a similar study where they assessed the attitudes of physicians regarding prenatal/antenatal oral health only half of the respondents advised the pregnant patients to visit the dentist however their reasoning was that they wanted the patients to delay the treatment until after pregnancy [23]. This highlights the importance of provider education regarding the role of healthcare professionals referring pregnant patients when necessary [24].

Developing an inter-professional relationship between healthcare professionals can improve communication and improve outcomes. This is especially beneficial consultations and the referral process. Through inter-professional communication, appropriate care for the prenatal/antenatal patients can be enhanced [25]. Unfortunately, in this study there was a lack of professional referral arrangements and consultations professionals’ discussions between the healthcare professionals with only 15.7% of the participants having professional referral arrangements and only 37.4% of them engaged in professional consultations. Similar findings were reported in another study regarding inter-professional collaboration between the healthcare professionals with only 14% of respondents having an established relationship with dental professionals for consultations and to provide referrals [16]. Increasing inter-professional collaboration would facilitate consultation and referral of pregnant patients to oral/dental care and improve outcomes for both mother and fetus.

Healthcare professionals have a responsibility for the oral care of the pregnant women. In particular, maternity care providers are the ones who see pregnant patients frequently especially for antenatal care. Therefore, it is very important to have understanding and knowledge regarding oral care in order to assess, educate and refer pregnant patients. Inconsistency in assessing oral health can be due to lack of knowledge in that area [18]. The results of this study indicated that the healthcare professionals were interested in enhancing their knowledge of oral care and most preferred to gain knowledge through Continuous Professional Development courses and inter-professional collaboration activities. Prior studies have also noted the importance of education and training on oral care for antenatal patients for healthcare professionals with healthcare professionals expressing understanding the importance of oral care during pregnancy, but lacking the skills and knowledge to provide advice to patients [20].

### Limitations

Because of the survey methodology design, response to the study was voluntary and relied on self-reporting and may not be representative of all maternity care providers in Brunei. Further, the study results are not globally generalizable although our results were similar to those found in other countries. The majority of respondents answered yes to the question “Are you interested in receiving additional information to enhance your knowledge regarding prenatal/antenatal oral healthcare?’ Future studies that include interventions to increase knowledge and skill are needed.

## Conclusion

The results from this study showed that most of the healthcare professionals had a generally positive attitude towards oral care of pregnant patients. Despite this, healthcare professionals often did not include oral care assessment as part of their routine practice during their patients’ assessments. Therefore, the findings of this survey study can be used to inform policy makers to prioritize oral care during perinatal period. Healthcare providers did indicate a desire to learn more about oral/dental care during pregnancy and this is an area that warrants future research.

## Data Availability

The datasets used for the current study are available from the corresponding author on reasonable request.
